# Antiseptics in Medical Cases

**Published:** 1897-06-19

**Authors:** 


### ANTISEPTICS IN MEDICAL CASES.

In the mind of the medical profession at large the words "antiseptic" or "aseptic" may be said to connote matters surgical, while their use as having an application to general medicine chiefly suggests an uncertain series of rites performed in connection with the acute specific fevers. Although certain diseases not belonging to the class of specific infectious fevers have been noted to occur in suggestive epidemics, and although clinical and pathological investigations point more and more to the microbial origin of such diseases and to the consequent indications for their prophylaxis and treatment, this indication has so far been little regarded in this country. In those hospitals and institutions where numbers of children are collected together, the frequent occurrence of the infectious fevers is regarded as a misfortune inherent in such places; while other morbid conditions to which children suffering from infectious fevers are so prone are, many of them, in their turn regarded as almost necessary features of such diseases, from which, however, they are entirely distinct. This latter is a singularly unfortunate point of view, seeing that it is so frequently to its secondary complications that a primary disease owes its deadly character. In most of our hospitals the prophylaxis consists in isolating those proved to be infected with an acute specific fever, together with subsequent measures of disinfection of a general character for the protection of the other patients, the method being in many cases equivalent to locking the stable door after the horse has been stolen, and being, moreover, liable to indefinite repetition.

Meantime, certain axioms are current at least in hospitals for children; for instance, that it is not advisable to admit any child unless it is said to have had measles and whooping-cough, that it is useless to take in various diarrhœas and wasting diseases with a view to doing them any good in the time at command, that various diseases of the respiratory organs "do better at home than in hospitals," and so on. In fact, in hospitals for children, the cases which it is desirable for their own good, or with due regard to others, to take in for treatment would seem to be decreasing rapidly. This difficulty is recognised, but the problem how, on the one hand, to isolate effectively those cases which are known to be infectious, and, on the other hand, to ascertain the kinds of disease of known or suspected microbic origin which may be safely treated together, seems altogether too hard for solution. In short, a working scheme of medical antiseptics is still to seek. Very clear ideas on this subject, however, seem to prevail at the Hospice des Enfants Assistés in Paris, and the very interesting measures of prophylaxis in force at this institution, in which a few years ago infectious diseases in their worst and most complicated forms were a positive scourge, are well worth studying. As a result of these measures not only did the infectious fevers themselves decline in a most remarkable way, but complications of every description did the same. Numerous complications became practically extinct, and the type of the original fevers was altered to one incredibly less severe, while the fall in the mortality became a matter of profound congratulation.

The difficulties to be contended with were very great. The hospice was old and badly adapted for its purpose, and the mortality from infectious diseases had always been very high. Thus, in the years 1867 to 1872, out of 1,256 children suffering from measles, 612, or 48 per cent., died — a mortality twice as great as that in the same period at the Trousseau Hospital. The cases were farther complicated by their association with diphtheria of a very malignant form. From 1886-91, in spite of all efforts (and good methods of sterilisation and disinfection were already in force), this had assumed such proportions that out of 235 cases there were 212 deaths—a mortality of 90 per cent.

The institution contains ordinarily about 600 children, a large number of these belonging to the poorest classes, and coming into the hospice during the illness or imprisonment of their parents. Only presumably healthy children are admitted. A significant fact was that, though infection was, in the first instance, brought to the hospice by new children, so that periodic outbreaks of measles following their admission occurred, there was a great preponderance of cases among those who were already inmates of the hospice. Thus, in 1891, out of 250 cases of measles, 22 only were admitted in the incubation stage of the disease; while 228 contracted it inside the hospice. The hospice thus proved itself a formidable source of danger to the healthy children admitted within its walls, and an efficient prophylaxis was urgently demanded if the institution was to justify its existence.

Professor Hutinel, who originated and directed the undertaking, satisfied himself that measles was not infectious during the incubation period, nor during convalescence. It was, on the other hand, intensely infectious from the beginning of the invasion up to two days before the eruption—a period of four full days. He determined that the most frequent mode of transmission was by direct contact or indirectly through contaminated objects, and very rarely by intermediary contact, except from bed to bed in the hospital wards. His first care was to secure a measure of quarantine for 15 days, through which each new child passed. This, on account of the limited room and impossibility of isolating the children in small enough groups, was not very efficiently carried out. Still it proved of great value in conjunction with the erection of a series of small isolation blocks in which cases of the acute specific fevers were separately dealt with. By these and other means not only were the cases of measles reduced to one-half, but other diseases fell in an equally remarkable way. Thus diphtheria in one year fell from 16 to three cases, whooping cough from 24 to 13 cases, scarlatina from 26 to three cases. An analysis of the high mortality showed that it was largely due to secondary infections. In 1891, among the cases of measles, 22 deaths were from diphtheria, while 56 were from broncho-pneumonia. Examination of the saliva of the patients and of the healthy children showed that staphylococci and streptococci were present twice as often in the former as in the latter. It seemed as if these organisms were rendered both extra-communicable and doubly active in the presence of infectious disease. And secondary infection was undeniable. Thus when a patient suffering from diphtheria plus broncho-pneumonia was introduced among cases of simple diphtheria isolated together, all contracted broncho-pneumonia. In this liability to secondary infection probably lay the reason why at the hospice as well as at the Trousseau Hospital the mortality rose as soon as a large collective isolation was practised, and fell again when only small carefully classified groups were isolated together. Under these latter conditions the mortality again fell, and two-thirds of the children suffering from measles survived. Such complications as stomatitis, laryngitis, otitis, conjunctivitis, and diarrhœa were found to be by no means incidental to the disease, and by the means taken were easily avoided. Pulmonary gangrene and ulcerative laryngitis, formerly common, became rare; noma and vulvar gangrene disappeared. In 1894, when the writer visited the hospice, two or three absolutely uninteresting infectious cases or suspects occupied the isolation blocks, while the measles and diphtheria wards stood empty. The fatality in 1895 stood at 16 per 100, 23 per 100 if subsequent death among tuberculous subjects was included.

After studying the remarkable results obtained in the Hospice des Enfants Assistés it remains to be seen in detail of what the general medical prophylaxis consists. The wards themselves are in some cases divided by glass partitions, each division containing a small number of beds, perhaps from one to twelve. Larger wards are divided down each side into "boxes," consisting of glazed partitions with doors, each containing two beds. The whole system of nursing is practically based on the idea that almost universal bed-to-bed infections are to be expected and guarded against. The nursing staff is a large one, and very close observation of each child is practised. Symptoms reckoned in any way suspicious are noted, and the subject of them at once isolated in a "box"—a delay of even a few hours being considered long enough to be dangerous to the other patients. Should an infectious fever manifest itself the case is then removed to the fever isolation huts. Sterilised blouses are worn by all the nursing staff and changed after contact with any "doubtful" case. Elaborate washing and disinfection of the hands is practised, and the routine daily sterilisation of all crockery, linen, &c., after use is carried out. Minute attention is paid to every possible source of contagion or contamination of the air or dust of the ward. All discharging eyes, ears, noses, and the smallest patches of impetigo, or peeling skin, or scurfy scalp, are covered with dressings or protective coverings. No case of bronchitis, diarrhœa, syphilis, skin disease, or sore throat is treated in a general ward. Such cases are isolated in the special "boxes" and carefully watched, or grouped in carefully classified groups in small wards. Cases of measles are isolated in small huts, each containing about six beds, simple cases and those presenting any complications being isolated separately. Each time the huts are empty of patients they are washed from floor to ceiling with corrosive sublimate solution, and the beds, &c., stoved. The room is then thrown open for several days to the air and sun. Not only is rigid cleanliness preserved, but antiseptics are in constant daily use, the ritual observed by the nursing staff being of the most thorough character. The patients on entering are given soap and water and disinfectant baths, while eyes, nose, mouth, and skin are subject to a routine inspection and disinfection. The secretions of the mouth are at intervals bacteriologically examined, and by this latter means cases of diphtheria are almost always diagnosed early before the appearance of the membrane. In the fever huts, as in the general wards, the appearance of a complication is a signal for a fresh and more rigorous isolation of the patient. A result which struck the writer as remarkable was the absence of the so-called sequelæ which are the common accompaniment of convalescence from the infectious fevers. These at the hospice were practically non-existent, or of the slightest character. It was claimed that this result was due to the above method of antiseptic treatment, a method which in this country would undoubtedly be called "meddlesome." The fashion, if so it may be called, of regarding asepsis as all-important and antiseptics as of little or no account is not, in Dr. Hutinel's view, based on entirely scientific principles. He uses antiseptics in every possible way in treating his patients. It is a matter for reflection that although surgically speaking an all-sufficient condition of asepsis can be maintained, medically speaking it cannot; and for the simple reason that in many cases, notably in the specific infectious febrile diseases, the patient himself is the centre and diffusing point of an infection which is co-existent with, and at every moment in active combat with, the surrounding asepsis. There is little analogy between a covered wound and a child with measles who is himself both the disseminator and the receiver of infection. The course taken by infectious disease when "left to itself" is only too clear from the history of the Paris Hospice. The course taken when half measures are employed is shown by the occurrence of the complications, mixed infections, and sequelæ which are all too common in our institutions for the treatment of infectious disease. Possibly the nature and *raison d'être* of our fever hospitals disposes to a pessimistic view of the diseases treated therein, whereas Dr. Hutinel, striving at the hospice in the cause of the healthy inmates and in the worst of conditions, was impelled perhaps to extraordinary measures in his crusade against infection. At any rate, in the light of his facts it seems as though in our hospitals our conceptions of the value of antisepsis might lend themselves to reconstruction.

